# 1744. Next generation sequencing (NGS) optimization of Oral Polio Vaccine samples

**DOI:** 10.1093/ofid/ofad500.1575

**Published:** 2023-11-27

**Authors:** Yuan J Carrington, Frank Zhou, Philip Tzou, Mingze Xiao, Sean Leary, Jonathan Altamirano, Yvonne A Maldonado

**Affiliations:** Stanford University School of Medicine, Stanford, California; Stanford University School of Medicine, Stanford, California; Stanford School of Medicine, stanford, California; n/a, sunnyvale, California; n/a, sunnyvale, California; Stanford University, Stanford, California; Stanford University, Stanford, California

## Abstract

**Background:**

As wild poliovirus is eradicated, preventing circulation of vaccine-derived poliovirus is top priority. Our lab developed real-time multiplex PCR and deep sequencing methods to detect and characterize OPV strains directly from stool samples. This method requires gel purification of PCR product created from viral RNA in stool samples. However, gel purification causes significant genetic material loss and is time consuming. Here, we explore combinations of different purification and NGS methods to optimize sequencing.

**Methods:**

554 OPV positive samples from a previous study were used. 991 isolates were identified: 268 serotype 1 (S1), 405 serotype 2 (S2), and 318 serotype 3 (S3). PCR amplicons created from viral RNA in stool were run through a 0.08% agarose gel to identity presence of the ∼3.5kb amplicon of interest. Amplicons then underwent 1 of 4 purification methods (Table 1) with those yielding DNA >10ng/ul sequenced using 1 of 2 NGS techniques (Miseq vs Nextseq).

Different Purification Methods Used Prior to NGS

**Figure:**
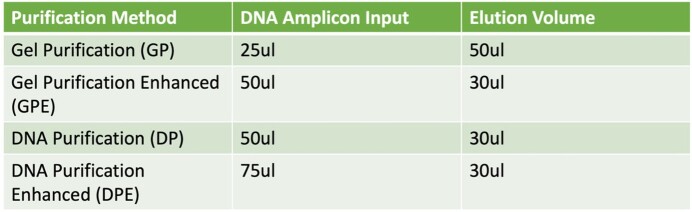

**Results:**

Gel purification: 42/126 (33.3%) S1 and 45/121(37.2%) S2 banded amplicons had a DNA concentration of >10ng/ul, as required for NGS. DNA purification: 449/450 (99.8%) S2 (259 banded) and all 318 (100%) S3 (170 banded) amplicons had concentrations >10ng/ul. S1 (n=145) samples had a higher mean coverage (MC) of 2850.02 than S2’s (n=208) MC of 792.56 and S3’s (n=217) MC of 256.78. Also, Nextseq (n=315) had a higher MC of 1486.78 than Miseq’s (n=255) MC of 648.99. Table 2 shows the MC for each purification and NGS method grouping and Graph 1 shows each grouping’s sample Ct value graphed against MC. 5/71 (7%) non-banded amplicons yielded sufficient sequencing coverage. There was a negative correlation between increasing Ct and coverage and a weak positive correlation between DNA concentration and coverage.

Coverage of OPV Positive Samples by Purification and NGS Methods

**Figure:**
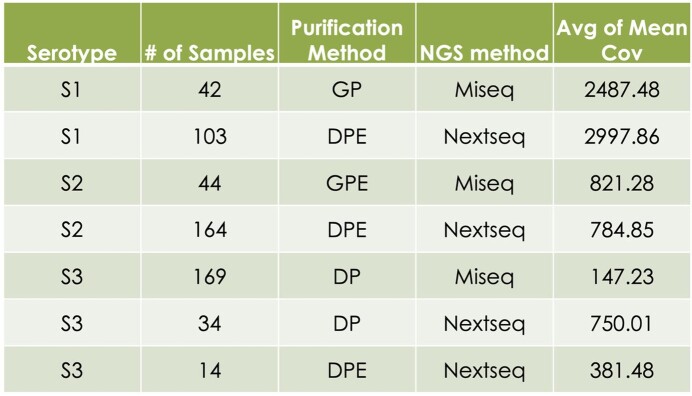

Ct Value Against Mean Coverage of Samples by Serotype and Purification and NGS Method Groups

**Figure:**
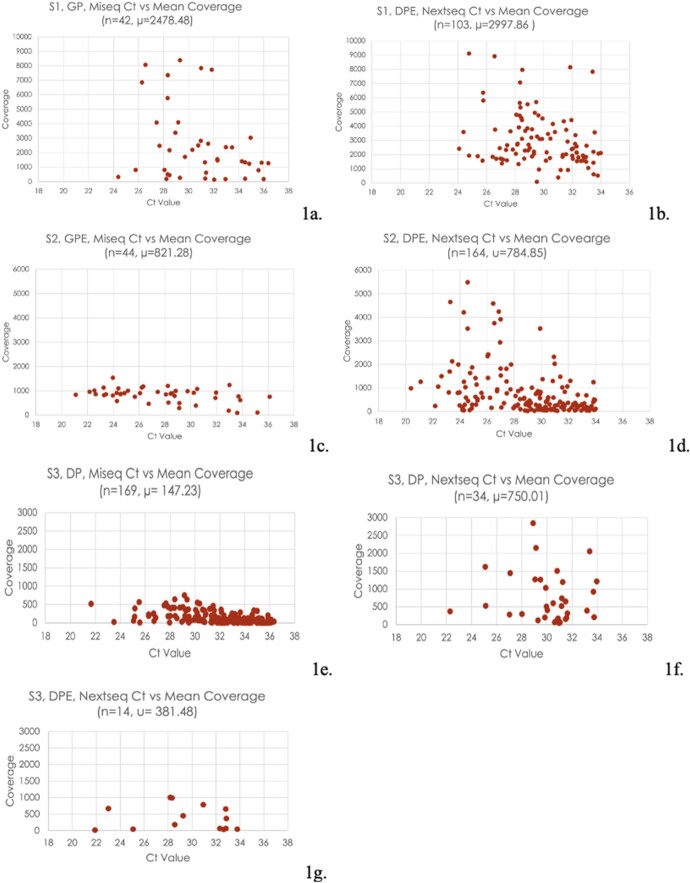

**Conclusion:**

DNA-purification methods (DPM) retain more DNA than gel-purification methods (GPM), therefore capturing a wider range of data. GPM may cause data loss and misrepresentation from reduction of sample size and variety, particularly of samples for within host variation analysis. However, the DNA retained via DPM were not OPV-specific, causing a need for a higher amplicon input volume to than GPM for the same coverage. DPM of band-producing samples with a Ct value of < 34 followed by Nextseq may be most optimal for NGS.

**Disclosures:**

**Yvonne A. Maldonado, MD**, Pfizer: Grant/Research Support|Pfizer: Site Investigator, DSMB member

